# Repeatedly measured predictors: a comparison of methods for prediction modeling

**DOI:** 10.1186/s41512-018-0024-7

**Published:** 2018-02-13

**Authors:** Marieke Welten, Marlou L. A. de Kroon, Carry M. Renders, Ewout W. Steyerberg, Hein Raat, Jos W. R. Twisk, Martijn W. Heymans

**Affiliations:** 1 Department of Epidemiology and Biostatistics, Amsterdam Public Health Research Institute, VU Medical Center, P.O. Box 7057, 1007 MB Amsterdam, The Netherlands; 2 Department of Public Health, Erasmus Medical Center, P.O. Box 2040, 3000 CA Rotterdam, The Netherlands; 3 Department of Health Sciences, Amsterdam Public Health Research Institute, VU University Amsterdam, De Boelelaan 1085, 1081 HV Amsterdam, The Netherlands; 4 Department of Medical Statistics and Bioinformatics, Leiden University Medical Center, P.O. Box 9600, 2300 RC Leiden, The Netherlands

**Keywords:** Statistical methods, Prediction models, Risk modeling, Longitudinal studies, Repeated measurements

## Abstract

**Background:**

In literature, not much emphasis has been placed on methods for analyzing repeatedly measured independent variables, even less so for the use in prediction modeling specifically. However, repeated measurements could especially be interesting for the construction of prediction models. Therefore, our objective was to evaluate different methods to model a repeatedly measured independent variable and a long-term fixed outcome variable into a prediction model.

**Methods:**

Six methods to handle a repeatedly measured predictor were applied to develop prediction models. Methods were evaluated with respect to the models’ predictive quality (explained variance *R*^2^ and the area under the curve (AUC)) and their properties were discussed. The models included overweight and BMI-standard deviation score (BMI-SDS) at age 10 years as outcome and seven BMI-SDS measurements between 0 and 5.5 years as longitudinal predictor. Methods for comparison encompassed developing models including: all measurements; a single (here: the last) measurement; a mean or maximum value of all measurements; changes between subsequent measurements; conditional measurements; and growth curve parameters.

**Results:**

All methods, except for using the maximum or mean, resulted in prediction models for overweight of similar predictive quality, with adjusted Nagelkerke *R*^2^ ranging between 0.230 and 0.244 and AUC ranging between 0.799 and 0.807. Continuous BMI-SDS prediction showed similar results.

**Conclusions:**

The choice of method depends on hypothesized predictor-outcome associations, available data, and requirements of the prediction model. Overall, the growth curve method seems to be the most flexible method capable of incorporating longitudinal predictor information without loss in predictive quality.

**Electronic supplementary material:**

The online version of this article (10.1186/s41512-018-0024-7) contains supplementary material, which is available to authorized users.

## Background

In recent years, the popularity of longitudinal studies has increased and with that the number of longitudinal studies itself [1]. These studies, in which participants are repeatedly measured over time, enable the monitoring of an individual evolution of disease or other (health-) outcome over time [1–3]. Numerous publications have been written on advanced statistical techniques for the analysis of repeated measurements as outcomes. However, in the literature, less emphasis has been placed on repeated measurements in independent variables.

Prediction models can be valuable tools for both public health and clinical practice [4]. They can assist in the identification of persons at high risk of being or becoming ill and estimate subject-specific probabilities of diagnostic and prognostic outcomes [4]. Most prediction models are characterized by the prediction of a fixed outcome measurement. Using repeated measurements of an independent variable would provide a model with more information about the variable’s trajectory or development over time than just a single measurement. This information could especially be interesting for the use in prediction modeling in circumstances in which not a single measurement but a certain trajectory of an independent variable has a strong association with the outcome, which may lead to a better prediction of this particular outcome. These improvements in individual risk estimation could in turn lead to improved health assessments and medical decision making in health care practice.

Few articles have been written on the various methods that could be applied for the analysis of longitudinally measured independent variables. The articles written by Chen et al. [5] and Tu et al. [6] discuss different methods that could be used for such analyses. However, their focus is not on the application of these methods for individual risk prediction, but on assessing growth trajectories and longitudinal exposure. Our aim is to compare different methods that can be used to model a repeatedly measured independent variable (longitudinal predictor) and a fixed outcome variable into prediction models and to evaluate the predictive quality of these models. We focused on methods that could relatively easily be applied by epidemiologists. Thus, for the current study, we selected available methods, that can handle longitudinal predictor information and a fixed outcome, are able to assess individual risk prediction, and are easily applied by epidemiologists. We excluded methods based on clustering and random effects as they are not appropriate for the use in individual prediction modeling. As an example we used data from the Terneuzen Birth Cohort [7] in which we applied various methods for the prediction of overweight and body mass index standard deviation score (BMI-SDS) at the age of 10 years with BMI-SDS measured between 0 and 5.5 years as a longitudinal predictor. In the discussion, the properties of the different methods are discussed with respect to the flexibility in handling missing data and differences in timing of measurements, the ability to incorporate all information of the longitudinal predictor, and to handle small or large numbers of repeated measurements, and user-friendliness.

## Methods

### Aim

To evaluate different methods to model a repeatedly measured independent variable and a long-term fixed outcome variable into a prediction model.

### Design and study population

The Terneuzen Birth Cohort consists of all 2604 children born in the city of Terneuzen between 1977 and 1986 [8]. Of the selected 2545 live-born children from singleton-births, 21 were excluded due to conditions possibly affecting growth. Another 941 children were excluded because they had less than two BMI measurements available in the age range of 0–5.5 years. For 853 children, there was no outcome variable of overweight and BMI-SDS at the age of 10 years available, leaving a total population for analysis of 730 children. The study protocol was approved by the Medical Ethics Committee of the VU University Medical Centre Amsterdam, and written informed consent was obtained from all participants [8].

### Data

Weight and length/height measurements with corresponding dates, which were prospectively registered by the Municipal Health Services, were retrieved for the Terneuzen Birth Cohort participants from birth onwards [8]. The weight and length/height measurements were used to calculate BMI (kg/m^2^). The exact age at each BMI measurement was calculated.

Next, all available BMI measurements were converted into BMI-SDS values as described by van Dommelen & van Buuren [9] using the LMS-method. The LMS-method is a method developed by Cole to generate smoothed growth curves by summarizing for each age the distribution of the growth data into LMS parameters (Lambda (L) skewness parameter; Mu (M) median; and Sigma (S) generalized coefficient of variation) and to calculate exact sds-scores from these LMS parameters [10–12]. The age-dependent BMI values from the Fifth Dutch Growth Study [13] were used as reference standard [9]. Because the BMI reference standard only contained (LMS-)values starting from 1 week onward for girls and from 2 weeks onward for boys, the reference standard values of the first two available weeks for boys and girls were linearly extrapolated to 0 weeks to enable the generation of BMI-SDS values at birth.

### Creating the example dataset

The growth data are organized in chronological order of visit per individual (e.g., weight at first visit, weight at second visit, etc.). The Preventive Child Health Care (CHC) Services aim for measurements to be conducted at predetermined ages for all children [14]. However, children often visit CHC Services at different ages because of planning problems of the parents or at the CHC organization and/or because of additional appointments beside the regular visits. It can therefore be that, for instance, the second available growth measurement for one child was taken at age 8 weeks, for another at age 2 years, and for yet another at the age of 7 years. Since the timing and number of the growth measurements differ considerably among children, adaptations to the data had to be made to construct a dataset with a more generic structure to be able to apply and compare all the different prediction methods.

#### The outcome variable

BMI-SDS at the age of 10 years is the outcome measurement and was computed by selecting the BMI measurement taken between the age range of 9–10.5 years for each subject and converting it into BMI-SDS (as previously described in Data). In case a subject had more than one BMI measurement available in this age range, the one measured closest to the aimed age of 10 years was selected. Additional to this continuous BMI-SDS outcome, a dichotomous outcome was created, using the International Obesity Task Force (IOTF) cut-offs for BMI to differentiate between non-overweight and overweight children at the age of 10 years [10, 15].

#### Longitudinal predictor

A longitudinal predictor was created with complete BMI-SDS values for each subject at seven specific ages, namely at birth/0 days, 3 months, 6 months, 14 months, 2 years, 3 years, and 5.5 years. These ages were chosen in order to resemble, as much as possible, the predefined ages of the regular visits used by CHC organizations. To create an example dataset with this longitudinal predictor the broken stick method was applied to all the available growth data from 0 to 5.5 years of the population for analysis to substitute missing BMI-SDS values while simultaneously generating BMI-SDS values at the specific ages [16]. The broken stick method uses a linear mixed model to describe subject specific growth trajectories at fixed times using a piecewise linear growth curve and is therefore a method that can be applied when the dataset contains irregular spacing of ages [16]. By the inclusion of random effects, subject-specific BMI-SDS values are obtained [16]. This example dataset with predictor BMI-SDS at the exact ages of 0 days, 3 months, 6 months, 14 months, 2, 3, and 5.5 years is referred to as the broken stick-data. See Additional file 1 for a visual representation of the differences in data structure between the original dataset and the newly generated broken stick-dataset. The development of the prediction models and their predictive quality assessments were performed in this broken stick-dataset. (Data available on request. The Terneuzen Birth Cohort data are not accessible in the public domain, because participants gave informed consent for retrieving information to be used for the Terneuzen project only.).

### Methods to develop prediction models

Different simple and slightly more advanced methods to handle a longitudinal predictor in a prediction model were considered for comparison and applied in the example dataset. The methods were derived from reviews of Chen et al. [5] and Tu et al. [6] who evaluated procedures to model individual trajectories and to analyze data with repeatedly measured independent variables and a non-time-varying outcome variable. For the current study we have chosen easily applicable methods that are able to handle a longitudinal predictor and a fixed outcome and are able to assess individual risk prediction.

Models were developed for the prediction of overweight (yes/no) using logistic regression and for the prediction of BMI-SDS at the age of 10 years using linear regression. In all developed prediction models, linear relationships between the predictor variables and the outcome were assumed. The following six methods were applied to develop prediction models with the repeatedly measured predictor BMI-SDS:

#### All measurements

In the first method all the seven measurements of BMI-SDS at the age of 0 days, 3 months, 6 months, 14 months, 2, 3, and 5.5 years were used as separate predictors in a multivariable regression analysis.

#### A single best measurement

The second method was a univariable analysis in which one of the seven age BMI-SDS measurements at 0 days, 3 months, 6 months, 14 months, 2, 3, or 5.5 years was chosen as the single “best” variable representing the predictor.

#### Summary measurement (mean or maximum)

This third method is similar to the second with a univariable analysis, but instead of choosing one of the measurements a summary measure, for example, the mean or maximum value of all the BMI-SDS measurements, is being used as the single variable representing the predictor in the model.

#### The change between subsequent measurements

A fourth method is to make use of the change between subsequent BMI-SDS measurements as predictors. Including the first BMI-SDS measurement at birth and all the six subsequent changes between BMI-SDS measurements in the model provides it with all the available information concerning a subject’s BMI-SDS growth trajectory [6].

#### The conditional measurements

A fifth method is to make use of conditional BMI-SDS measurements. A conditional measurement of a certain time point is obtained by taking the (BMI-SDS) measurement of that specific time point and regress all previous measurements on it [6]. The residual of this equation is the conditional (BMI-SDS) measurement of the specific time point uncorrelated to the previous measurements [6]. Using the first original (BMI-SDS) measurement at birth and all subsequent conditional (BMI-SDS) measurements provides the model with all the available information concerning a subject’s (BMI-SDS) growth trajectory [6].

#### Growth curve parameters of BMI-SDS over time

In the sixth method, we performed a two-step analysis based on information derived from a child’s individual growth curve. First, a linear regression analysis is performed with age at measurement as independent variable and the BMI-SDS measurement as the dependent variable. This regression analysis is performed with the growth data from 0 days to 5.5 years for each subject separately in a long structured dataset to obtain subject specific growth curves over this time interval. Apart from a linear growth curve, we also applied quadratic and cubic functions.

Secondly, the mean of all measurements and the child-specific growth parameters, i.e., slope coefficient(s)) are entered in a new model to predict the outcome overweight and BMI-SDS at 10 years, again assuming a linear association between the predictor(s) and the outcome as for the previous methods. Here the mean represents the individual’s average value of BMI-SDS from 0 to 5.5 years and the slope parameters [5, 17–20] represent the trend of the individual’s change in BMI-SDS over time. We also added the standard error of the slope parameter from the first step to the model as an indication of fluctuation of the actual measurements around the curve [17–20].

### Performance assessment of the prediction models

The predictive quality of the prediction models was assessed with several performance measures. Discrimination of the prediction models, i.e., the ability to differentiate between persons who do and do not develop the outcome, can be assessed with the explained variance *R*^2^ and the area under the curve (AUC) [21, 22]. For the logistic models adjusted Nagelkerke *R*^2^, as described by Steyerberg [4], was used and adjusted *R*^2^ for the linear models. The AUC was used for both the logistic and linear models, with the outcome of *observed* overweight (yes/no) at the age of 10 years as the state variable and the predicted risk for overweight (logistic model) or predicted BMI-SDS value (linear model) at 10 years as test variable. The DeLong method [23] was used to test for significant differences between AUC-values.

All analyses were performed using the Statistical Package for the Social Sciences version 22.0 for Windows (SPSS Inc., Chicago, IL, USA). The broken stick and DeLong method were applied using R for Windows version 3.2.5 (The R Foundation).

## Results

### Population for analysis

**Fig. 1:**
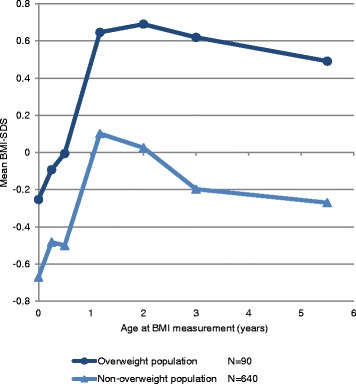
Mean BMI-SDS at ages 0 to 6 years of overweight and non-overweight 10-year-old children

The characteristics of the population for analysis are shown in Table 1. Around the age of 10 years (median age 9.9 years, 95% range 9.1, 10.4) mean BMI-SDS was −  0.2 SDS (standard deviation 1.0) and 90 participants (12.3%) were overweight according to the IOTF cut-offs. Figure 1 shows the development in mean BMI-SDS over time (age 0 days to 5.5 years) of the non-overweight and the overweight group at the age of 10 years.

**Table 1:** Characteristics of the population for analysis

		Broken stick-data *N* = 730
	Male, no (%)	343 (47.0%)
Visit 0 days	Age (years)	0.0 (0.0; 0.0)
	BMI-SDS	−0.6 (0.9)
Visit 3 months	Age (years)	0.3 (0.3; 0.3)
	BMI-SDS	−0.4 (0.8)
Visit 6 months	Age (years)	0.5 (0.5; 0.5)
	BMI-SDS	−0.4 (0.8)
Visit 14 months	Age (years)	1.2 (1.2; 1.2)
	BMI-SDS	0.2 (0.8)
Visit 2 years	Age (years)	2.0 (2.0; 2.0)
	BMI-SDS	0.1 (0.8)
Visit 3 years	Age (years)	3.0 (3.0; 3.0)
	BMI-SDS	−0.1 (0.8)
Visit 5.5 years	Age (years)	5.5 (5.5; 5.5)
	BMI-SDS	−0.2 (0.7)
Visit 10 years	Age (years)	9.9 (9.1; 10.4)
	BMI-SDS	−0.2 (1.0)
	Overweight, no (%)	90 (12.3%)

Values are expressed as the mean (SD), median (95% range) or number (%) of age at visit, BMI standard deviation score (-SDS) at visit, sex, and overweight

### Predictive quality of the prediction models

The predictive quality of the prediction models that were developed using the six different methods are shown in Table 2.

**Table 2:** The predictive quality of prediction models developed using different methods to include longitudinal predictor BMI-SDS

Method	Model includes	Outcome at 10y			
		Overweight		BMI-SDS	
		Nk R^2^	AUC	*R* ^2^	AUC
1. All original measurements	BMI-SDS at age 0 days, 3 months, 6 months, 14 months, 2 years, 3 years, 5.5 years	0.244^a^	0.807^a^	0.339^b^	0.801^b^
2. Single ‘best’ measurement	BMI-SDS at age 5.5 years	0.230	0.799	0.329	0.799
3. Summary measurement	Mean (BMI-SDS at age 0 days, 3 months, 6 months, 14 months, 2 years, 3 years, 5.5 years)	0.168	0.767	0.238	0.767
3. Summary measurement	Maximum (BMI-SDS at age 0 days, 3 months, 6 months, 14 months, 2 years, 3 years, 5.5 years)	0.130	0.737	0.177	0.737
4. Change between measurements	BMI-SDS at age 0 days and BMI-SDS changes between ages 3_m_-0_d_, 6_m_-3_m_, 14_m_-6_m_, 2_y_-14_m_, 3_y_-2_y_, 5.5_y_-3_y_	0.244^c^	0.807^c^	0.339^d^	0.801^d^
5. Conditional measurements	BMI-SDS at age 0 days and conditional BMI-SDS at age 3 months, 6 months, 14 months, 2 years, 3 years, 5.5 years	0.244^e^	0.807^e^	0.348	0.806
6. Growth curve parameters	Mean and regression coefficients of the cubic growth curve ($mean,{b}_{age},{b}_{age^2},{b}_{age^3}$)	0.241	0.803	0.337	0.803

Values are the explained variance of each prediction model developed in the broken stick dataset expressed in adjusted Nagelkerke *R*^2^ (Nk *R*^2^) or adjusted *R*^2^ (*R*^2^) and the area under the curve (AUC). The models predicting the dichotomous outcome overweight no/yes were analyzed using logistic regression. The prediction models predicting the continuous outcome BMI-SDS at age 10 were analyzed using linear regression

Due to collinearity: a. The model did not contain BMI-SDS at 5.5 years; b. The model did not contain BMI-SDS at 3 years; c. The model did not contain ΔBMI-SDS between 5.5_y_-3_y_; d. The model did not contain BMI-SDS at 0 days; e. The model did not contain conditional BMI-SDS at 5.5 years

This table shows that methods 1 “all measurements”; 2 “single best measurement”; 4 “changes between measurements”; 5 “conditional measurements”; and 6 “growth curve parameters” were the ones that produced logistic models for the prediction of overweight at age 10 years of the highest predictive quality: with adjusted Nagelkerke *R*^2^-values of 0.230 to 0.244 and AUC-values of 0.799 to 0.807. The 5.5 year BMI-SDS (method 1 and 5) and the 5.5_y_-3_y_ BMI-SDS change (method 4) measurement were automatically removed from these models as a result of collinearity, i.e., these variables did not bring any additional information to the model and were therefore of no extra value. Only the AUC-values 0.767 and 0.737 of method 3 “summary measurements” using the mean and median were significantly different from the AUC-value 0.807 of reference method 1 (*p* values ≤ 0.031).

For the logistic model of method 2 the 5.5y BMI-SDS measurement was used as the single “best” predictor as it produced the model of the highest predictive quality. The effect of choosing one of the other measurements as single “best” predictor is shown in Additional file 2: Table S3. The table shows a general trend of an increase in predictive quality of the model when increasing age of BMI-SDS measurement with a significantly lower AUC for the measurements from 0 to 2 years compared to the reference 5.5 year measurement (*p* values ≤ 0.001).

In the logistic model of method 6 “growth curve parameters” we used the model based on individuals’ cubic growth curves for comparison as this produced the model with the highest predictive quality. The predictive quality of the models developed with linear and quadratic growth curves and models including the standard error are presented in Additional file 2: Table S4. Overall the models’ predictive quality improved when a quadratic instead of a linear growth curve was used; remained roughly similar when a cubic instead of a quadratic growth curve was used; and did not seem to improve by the inclusion of the standard error. However, none of the AUC’s of the different growth curves were statistically significantly different from the AUC of the model including the cubic growth curve parameters and standard error.

Table 2, Additional file 2: Table S3, and S4 also show the results for the linear models predicting BMI-SDS at the age of 10 years. The results of these linear models were similar to the results of the logistic models described above with as main result that methods 1, 2, 4, 5, and 6 showed the highest predictive performance.

## Discussion

In this article, we applied six relatively simple methods to develop a prediction model with a fixed outcome and a repeatedly assessed longitudinal predictor. Using these methods, we developed prediction models for overweight and BMI-SDS at the age of 10 years (including the repeatedly measured predictor BMI-SDS age 0–5.5 years) and evaluated their predictive quality. Overall, the models developed by the different methods were of similar predictive quality, apart from the models of method 3 “summary measurement” which were of lower predictive quality.

### Methods to model a longitudinal independent variable

Most prediction models are developed without the inclusion of longitudinal predictor information. Including this information, however, could lead to improvement of individual risk prediction. There are various methods to include a longitudinally measured independent variable into a model, which are discussed in overviews by Chen et al. [5] and Tu et al. [6]. However, most of these methods are not primarily designed for the purpose of risk prediction. For the current study we selected methods that can handle longitudinal predictor information and a fixed outcome, are able to assess individual risk prediction, and are easily applied by epidemiologists. Methods that we have not discussed are for example methods based on clustering such as Gaussian mixture model by clustering the exposure values [5]; functional clustering models [5]; and functional logistic regression model [5]; and methods based on random effects such as latent growth curve modeling [6]; and growth mixture modeling [6]. The clustering methods are based on the grouping of individual trajectories leading to a loss of information and are difficult to use for individual risk prediction as it is unknown to which group a new individual belongs in practice. Methods based on random effects are not appropriate for the use in prediction modeling as in practice the random effect(s) of an individual are unknown and, therefore, only the fixed effects can be used (which comes down to the growth curve method described in this paper). Another method that receives attention is the use of joint modeling [24]. This method is appropriate for longitudinal data in combination with survival data and therefore fell beyond the scope of this article. Joint modeling loses its surplus value when there is no survival data but an outcome measured at a fixed time-point after the last predictor measurement. This is because the last estimated predictor measurement before the occurrence of an event is used for the final model, which would be the estimated 5.5y BMI-SDS measurements here (coming down to method 2 with actual 5.5y BMI-SDS). At last, there is the method of curve matching [25], a new method that would be suitable and could be interesting for individual risk prediction. However, curve matching is still under development and we were therefore unable to include it in our comparison.

### Methods to develop prediction models with a longitudinal predictor

At first sight one might consider method 1 “all measurements” to be the most straightforward method. Its advantage is that it is easily applied and that all of the available information on the predictor is being used [5, 6]. A major limitation is that it requires for all subjects to be measured around similar time points [5, 6] and to have no missing values [5] for any of the measurements; otherwise, no prediction can be made. Another disadvantage of this method is the risk of running into the problem of collinearity [5, 6], causing some of the predictor’s variables to be removed from the model. Finally, a limitation is that more variables have to be entered in the model when more measurements are available. This is problematic with a large number of repeated measurements as it is desirable to end up with a good yet parsimonious prediction model that is easily applicable in practice.

An advantage of method 2 “single best measurement” and method 3 “summary measurement” is that they are also simple to apply and there is no problem with collinearity [5]. An important limitation of these methods is the loss of longitudinal predictor information. Another limitation is that method 2 “single best measurement” is not flexible with the timing of the measurements and handling of missing values. Method 3 “summary measurement”, however, does not require availability of all measurements to calculate a mean or maximum of the available data and is therefore somewhat more flexible with the timing and missing values of measurements.

An advantage of method 4 “changes between measurements” and method 5 “conditional measurements” is that they use all available information on an individual’s growth development and can handle the problem of collinearity slightly better than method 1 “all measurements” [6]. However, as was seen in the example, collinearity might still occur. A limitation of these methods is that many variables will have to be included in the model if there are many repeated measurements and the methods are not flexible in the presence of missing data. However, some flexibility in the timing of the measurements is possible for method 4 “changes between measurements” as a sort of ratio of change could be used (i.e., change divided by the covered period of time), but these ratios should still cover approximately the same age ranges. Moreover, method 5 “conditional measurements” requires participants to be measured at similar time points [6].

The advantage of method 6 “growth curve parameters” is that it can handle the problem of collinearity while taking all information on a subject’s growth into account. When there are many repeated measurements only a couple of variables need to be entered in the prediction model, as all original measurements are summarized into the mean and a (few) growth curve parameter(s) at hardly any cost to the predictive quality of the model. Another advantage is the flexibility of the model with missing values and timing of measurements; this method can even be employed if people are irregularly measured over time. A down side of this method is that it needs to be performed in two steps and you need to import the results of the analyses of step 1 into your dataset before performing step 2, thus it is a slightly less straightforward method. Moreover, this method might not be useful in circumstances where the predictor only has a very small (2-3) number of repeated measurements, because you will end up having to enter the same number of variables into the prediction model while having a loss of data due to simplifying the data into a growth curve parameter.

See Table 3 for a quick overview of the pros and cons of each method.

**Table 3:** Characteristics of the methods for developing a prediction model with a longitudinal predictor

Method	Flexible with missing values	Flexible with timing of measurements	Encompasses all information on the development of the predictor	Capable of dealing with a great number of repeated measurements	Capable of dealing with a small number of repeated measurements	Straightforward predictor computation (no additional steps that need to be performed before prediction model can be made)
1. All original measurements			+		+	+
2. Single “best” measurement				+	+	+
3. Summary (mean or maximum etc.)	+	+		+	+	*
4. Change between measurements		*	+		+	*
5. Conditional measurements			+		+	*
6. Growth curve parameters	+	+	+	+		

^+^advantage that is present; *advantage that is partially present; an empty cell indicates an advantage that is not present. See discussion section "Methods to develop prediction models with a longitudinal predictor" for more information

### Strengths and limitations

For this study we used data from the Terneuzen Birth Cohort and created a complete example dataset with seven repeated measurements at specific time points. Unfortunately, the Terneuzen data contained many missing values and applying the broken stick method could possibly have led to too much coherence between subsequent measurements causing collinearity. However, body size measurements that succeed each other tend to be correlated [6]; therefore, collinearity is not uncommon when repeated growth measurements are included in a single model. Because it could not be ruled out that using the broken stick method had its own effect on the performance of the different methods, we performed the same analyses in another example dataset which was constructed by restructuring the Terneuzen data and applying multiple imputations. Results of these analyses regarding the different methods were similar to those discussed in the previous results section. See Additional file 2: Text S1 for further details on how these analyses were performed and Additional file 2: Figure S2 and Table S1-S4 for the replicated results by these analyses.

We believe that we have provided a clear overview of the most relevant methods for epidemiologists and clinicians to consider for individual risk prediction with longitudinal predictors. A strength of this study is its focus on the applicability of the different methods for epidemiologists. When the appropriate method is applied, prediction models can make optimal usage of the available information provided by the repeatedly measured predictors. The improved predictions from these models may subsequently lead to improved decision making by clinicians in health care settings. Moreover, measuring a predictor repeatedly enables health care professionals to recalculate a client’s risk for a specific outcome at each new visit and to adjust the course of a treatment if needed. Further research is needed to determine how this should be realized.

### Practical implications

Having discussed the different methods, the question of which method to apply for the development of a prediction model for daily practice with a longitudinal predictor rises. Unfortunately, there is not a single straightforward answer. All methods are considered appropriate for different situations. The choice of method will depend on (1) the hypothesized association between predictor and outcome, (2) the available data that will be used to construct the prediction model, and (3) the requirements of the prediction model for use in practice. Each of the following criteria must be considered:

#### Relation between predictor and outcome

When the developmental curve of the predictor is of importance for the risk estimation on the outcome, all methods described in this article can be used apart from the univariable methods 2 “single best measurement” and 3 “summary measurement” as the loss of information on the predictor’s development would lead to a decreased predictive quality. Method 6 “growth curve parameters” would be suitable when it is expected that there are distinct developmental trajectories of the predictor across outcome groups and that these trajectories are adequately reflected by the growth curve parameters [5]. When it is well known that a specific measurement is the most optimal predictor, method 2 “the best measurement” could be used. The choice of which single variable you choose needs to be soundly-based to justify its use as the “best” as it assumes that the single measurement is representative for, or even has superior predictive value compared to, all the other measurements and that its development over time is not of importance for the predictive accuracy. The use of method 3 with the mean of all measurements would be more appropriate when there is no change in the predictor over time or when changes are not associated with the outcome [5]. When an acute or extreme occurrence of exposure to the predictor is associated with the outcome, and not the development of the predictor over time or its mean level, the use of method 3 with the maximum value could be considered as a suitable method [5].

#### Missing data

Another important property to consider is a method’s capability to handle missing data. Method 1 “all measurements”, method 4 “change between measurements”, and method 5 “conditional measurements” require that there are no missing values in any of the variables, as all variables need to be entered in the model to generate an individual’s prediction. These methods might therefore be less appropriate for developing prediction models to be used in settings where missing values regularly occur, although one could also apply a proper method of imputing these missing values to still generate predictions. Method 3 “summary measurement” and method 6 “growth curve parameters” are more flexible and can still be used to calculate predictive values when a person has a missing value for a (couple of) measurement(s). However, although these methods are flexible in the handling of missing values a sufficient amount of measurements is still needed to make reliable predictions.

#### Data structure

Most of the time, repeated measurements are organized in variables within certain time-intervals. In this case, all methods could be applied. However, when this is not the case and measurements are irregularly taken over time, method 4 “change between measurements” might be applied when there are only small differences in timing of measurements. When this is the case, the ratio of change could be calculated over certain periods. Methods 3 “summary measurement” and 6 “growth curve parameters” are flexible and do not require the data to be measured at specific time points. However, despite the flexibility of the methods the input data still needs to cover the same data construct used to develop the model and give enough information to cover the development of the predictor over the aimed period of time to make reliable estimates.

#### Number of repeated measurements

The number of repeated measurements also affects the choice of method. When there are only two or three repeated measurements of the predictor, method 6 “growth curve parameters” may be redundant. However, when there are a great number of repeated measurements it is no longer feasible to put all the BMI-SDS measurements, the change or the conditional measurements in a prediction model (method 1, 4, and 5). Method 6 could then be used to summarize all these values into a growth curve described by two to three variables. Methods 2 “single best measurement”, 3 “summary measurement” could also be used in case of many repeated measurements, but these methods are much less refined as information on the development of the predictor is lost.

#### Computational cost

As stated before we have focused on comparing prediction methods that are easily applicable for epidemiologists, as all the 6 methods are easily applied none of the methods should probably be selected or rejected based on computational costs: they are all very manageable for statistical software programmes if applied appropriately. Although there are differences in computational costs for the different methods these are very difficult to quantify as it will depend on different factors such as the structure (current and desired for analysis) and size (number of persons; number of measurements; and the number of imputed datasets if applicable) of the dataset.

## Conclusion

The choice of method for developing a prediction model with a longitudinal predictor depends on hypothesized predictor-outcome associations, available data, and requirements of the prediction model. For this article’s example, predicting overweight at age 10 years with longitudinal predictor BMI-SDS, method 6 “growth curve parameters” is probably the best choice. This is because (1) we expect that children’s BMI-SDS development over the ages 0–5.5 years is associated with overweight at age 10 years [26]; (2) missing values and irregular measurements occur regularly in CHC practice; and (3) a considerable amount of repeated BMI-SDS measurements are available.

The growth curve method is the one that is best capable to encompass all information on development while still being able to handle missing values and irregular measurements in a flexible manner. Moreover, with this method irregularly measured data would not have to be restructured in a generic structured dataset to be able to build a prediction model. Therefore, when dealing with a predictor that has been measured for considerable times and its development over time is expected to be associated with the outcome, the growth curve method seems to be the method best to be applied and used in practice.

## Additional files

Additional file 1: Figure showing the structure of the original Terneuzen Birth Cohort data, the broken stick data, and the multiple imputed-data; Timing of BMI-SDS measurement for five selected participants of the Terneuzen Birth Cohort. (PDF 35 kb)

Additional file 2: Information on (Text S1) and results of (Figure S2 and Tables S1-4) the analyses reproduced in the multiple imputed dataset. (DOCX 45 kb)

## Abbreviations

AUC: Area under the curve
BMI: Body mass index
CHC: Child Health Care
IOTF: International Obesity Task Force
SDS: Standard deviation score

## References

[1] Lynn P. Methodology of longitudinal surveys. Chichester: Wiley; 2009.https://books.google.nl/books?hl=nl&lr=&id=ixtAyTOc2FkC&oi=fnd&pg=PR5&dq=Lynn+P:+Methodology+of+Longitudinal+Surveys.+Wiley%3B+2009.&ots=PZgmRDJdUG&sig=jmstidEw8HzMTR8K_2XK8oygEsg#v=onepage&q=Lynn%20P%3A%20Methodology%20of%20Longitudinal%20Surveys.%20Wiley%3B%202009.&f=false

[2] Twisk JWR. Applied longitudinal data analysis for epidemiology: a practical guide. Cambridge: Cambridge University Press; 2003. https://books.google.nl/books?id=TCg02e-tI_cC&printsec=frontcover&hl=nl&source=gbs_ge_summary_r&cad=0#v=onepage&q&f=false

[3] Fitzmaurice GM, Laird NM, Ware JH: Applied Longitudinal Analysis. Hoboken: Wiley; 2012. https://books.google.nl/books?id=0exUN1yFBHEC&printsec=frontcover&hl=nl&source=gbs_ge_summary_r&cad=0#v=onepage&q&f=false

[4] Steyerberg E: Clinical prediction models: a practical approach to development, validation, and updating. New York: Springer Science+Business Media; 2008.https://books.google.nl/books?hl=nl&lr=&id=kHGK58cLsMIC&oi=fnd&pg=PR2&dq=Steyerberg+E:+Clinical+Prediction+Models:+A+Practical+Approach+to+Development,+Validation,+and+Updating&ots=TNTfDYbGqo&sig=P24KsicEGWDBJjLACSSWI1KaAxo#v=onepage&q=Steyerberg%20E%3A%20Clinical%20Prediction%20Models%3A%20A%20Practical%20Approach%20to%20Development%2C%20Validation%2C%20and%20Updating&f=false

[5] Chen YH, Ferguson KK, Meeker JD, TF ME, Mukherjee B (2015). Statistical methods for modeling repeated measures of maternal environmental exposure biomarkers during pregnancy in association with preterm birth. Environ Health.

[6] Tu YK, Tilling K, Sterne JA, Gilthorpe MS (2013). A critical evaluation of statistical approaches to examining the role of growth trajectories in the developmental origins of health and disease. Int J Epidemiol.

[7] de Kroon ML, Renders CM, Kuipers EC, van Wouwe JP, van Buuren S, de Jonge GA, Hirasing RA (2008). Identifying metabolic syndrome without blood tests in young adults--the Terneuzen birth cohort. Eur J Pub Health.

[8] de Kroon ML, Renders CM, van Wouwe JP, Hirasing RA, van Buuren S (2011). Identifying young children without overweight at high risk for adult overweight: the Terneuzen birth cohort. Int J Pediatr Obes.

[9] van Dommelen P, van Buuren S: Documentatie berekening Standaarddeviatiescore. TNO; 2011. https://repository.tudelft.nl/view/tno/uuid:3db2d004-ff85-45cf-8cf0-8ac54588f894/

[10] Cole TJ, Bellizzi MC, Flegal KM, Dietz WH (2000). Establishing a standard definition for child overweight and obesity worldwide: international survey. BMJ.

[11] Cole TJ, Freeman JV, Preece MA (1995). Body mass index reference curves for the UK, 1990. Arch Dis Child.

[12] Flegal KM, Cole TJ: Construction of LMS parameters for the Centers for Disease Control and Prevention 2000 growth charts. US Department of Health and Human Services, Centers for Disease Control and Prevention, National Center for Health Statistics; 2013.

[13] Schonbeck Y, Talma H, van Dommelen P, Bakker B, Buitendijk SE, Hirasing RA, van Buuren S (2011). Increase in prevalence of overweight in Dutch children and adolescents: a comparison of nationwide growth studies in 1980, 1997 and 2009. PLoS One.

[14] (JGZ) PJ (2003). Richtlijn Contactmomenten Basistakenpakket Jeugdgezondheidszorg 0-19 jaar.

[15] Cole TJ, Lobstein T (2012). Extended international (IOTF) body mass index cut-offs for thinness, overweight and obesity. Pediatr Obes.

[16] van Buuren S: Flexible imputation of missing data*.* Boca Raton: CRC Press, Taylor & Francis Group, LLC; 2012.https://books.google.nl/books?hl=nl&lr=&id=M89TDSml-FoC&oi=fnd&pg=PP1&dq=Buuren+S:+Flexible+Imputation+of+Missing+Data.+Taylor+%26+Francis%3B+2012&ots=BcqTjiPtlh&sig=BIsE_PEIJijBfVs8Yfsdihp1nTA#v=onepage&q&f=false

[17] Zhang H, Tamakoshi K, Yatsuya H, Murata C, Wada K, Otsuka R, Nagasawa N, Ishikawa M, Sugiura K, Matsushita K (2005). Long-term body weight fluctuation is associated with metabolic syndrome independent of current body mass index among Japanese men. Circ J.

[18] Tamakoshi K, Yatsuya H, Kondo T, Ishikawa M, Zhang H, Murata C, Otsuka R, Mabuchi T, Hori Y, Zhu S (2003). Long-term body weight variability is associated with elevated C-reactive protein independent of current body mass index among Japanese men. Int J Obes Relat Metab Disord.

[19] van de Langenberg D, Hoekstra T, Twisk JW, van Wouwe JP, Hirasing RA, Renders CM, de Kroon ML (2015). Weight fluctuation during childhood and cardiometabolic risk at young adulthood. J Pediatr.

[20] Lee JS, Kawakubo K, Kobayashi Y, Mori K, Kasihara H, Tamura M (2001). Effects of ten year body weight variability on cardiovascular risk factors in Japanese middle-aged men and women. Int J Obes Relat Metab Disord.

[21] Altman DG, Vergouwe Y, Royston P, Moons KG (2009). Prognosis and prognostic research: validating a prognostic model. BMJ.

[22] Royston P, Moons KG, Altman DG, Vergouwe Y (2009). Prognosis and prognostic research: developing a prognostic model. BMJ.

[23] ER DL, DM DL, Clarke-Pearson DL (1988). Comparing the areas under two or more correlated receiver operating characteristic curves: a nonparametric approach. Biometrics.

[24] Asar O, Ritchie J, Kalra PA, Diggle PJ (2015). Joint modelling of repeated measurement and time-to-event data: an introductory tutorial. Int J Epidemiol.

[25] van Buuren S (2014). Curve matching: a data-driven technique to improve individual prediction of childhood growth. Ann Nutr Metab.

[26] Stocks T, Renders CM, Bulk-Bunschoten AM, Hirasing RA, van Buuren S, Seidell JC (2011). Body size and growth in 0- to 4-year-old children and the relation to body size in primary school age. Obes Rev.

