# Supplementary material for: Repeatedly measured predictors: a comparison of methods for prediction modeling
Source: Diagn Progn Res. 2018 Feb 13;2:5. doi: 10.1186/s41512-018-0024-7 (PMC6460730; doi:10.1186/s41512-018-0024-7)
Supplement: Supplementary file 1 — Figure showing the structure of the original Terneuzen Birth Cohort data, the broken stick data, and the multiple imputed-data; Timing of BMI-SDS measurement for five selected participants of the Terneuzen Birth Cohort. (PDF 35 kb) [file 41512_2018_24_MOESM1_ESM.pdf]

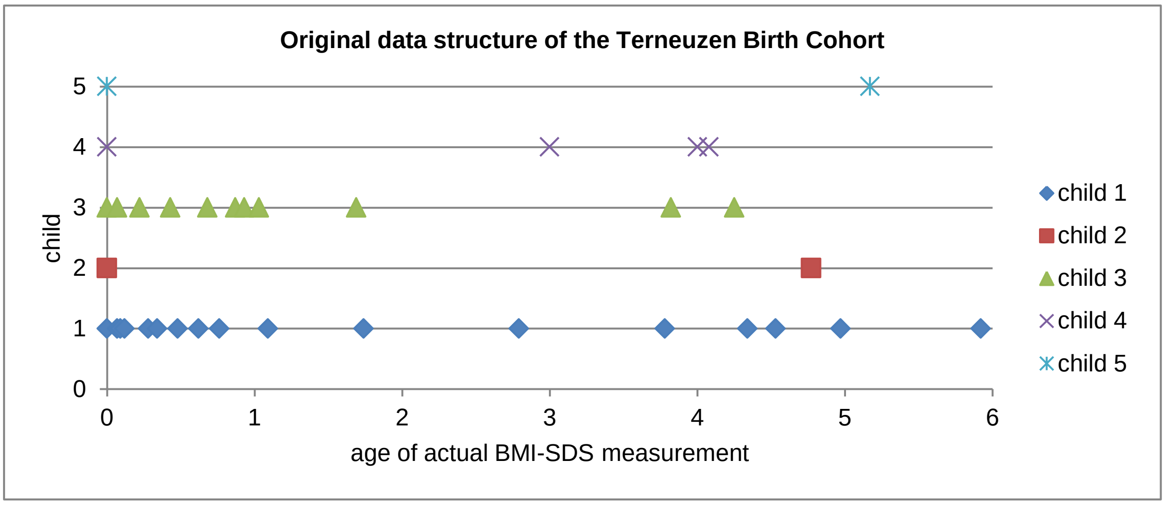


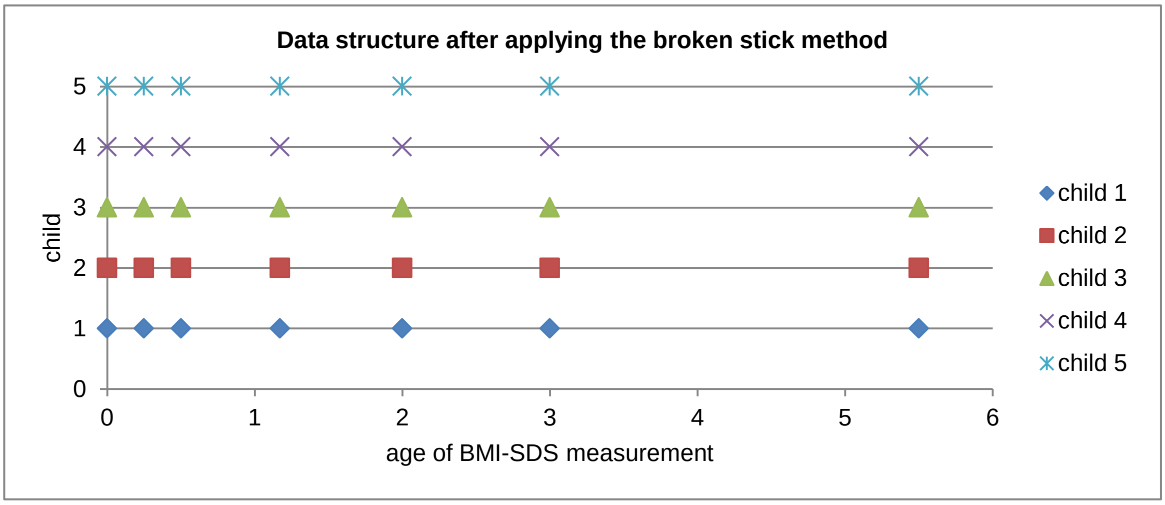


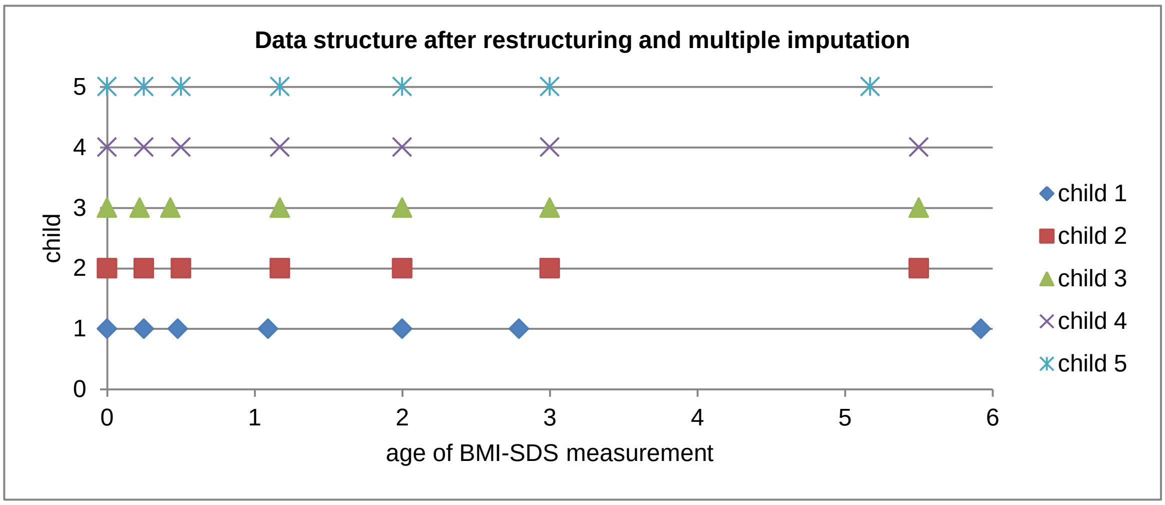


**Additional file 1.** The structure of the original Terneuzen Birth Cohort data, the broken stick data, and the multiple imputed-data; Timing of BMI-SDS measurement for five selected participants of the Terneuzen Birth Cohort.
