# Supplementary material for: Repeatedly measured predictors: a comparison of methods for prediction modeling
Source: Diagn Progn Res. 2018 Feb 13;2:5. doi: 10.1186/s41512-018-0024-7 (PMC6460730; doi:10.1186/s41512-018-0024-7)
Supplement: Supplementary file 2 — Information on (Text S1) and results of (Figure S2 and Tables S1-4) the analyses reproduced in the multiple imputed dataset. (DOCX 45 kb) [file 41512_2018_24_MOESM2_ESM.docx]

**Additional file 2**

**Repeatedly measured predictors: a comparison of methods for prediction modeling**

Marieke Welten*^1^, M.Sc, Marlou L.A. de Kroon^2^, MD, PhD, Carry M. Renders^3^, PhD,

Ewout W. Steyerberg^2,4^, PhD, Hein Raat^2^, MD, PhD, Jos W.R. Twisk^1^, PhD, Martijn W. Heymans^1^, PhD.

^1^ Department of Epidemiology and Biostatistics, Amsterdam Public Health Research Institute, VU Medical Center, P.O. Box 7057, 1007 MB Amsterdam, The Netherlands. E-mail: m.welten@vumc.nl, jwr.twisk@vumc.nl, mw.heymans@vumc.nl

^2^ Department of Public Health, Erasmus Medical Center, P.O. Box 2040, 3000 CA Rotterdam, The Netherlands. E-mail: m.dekroon@erasmusmc.nl, e.steyerberg@erasmusmc.nl, h.raat@erasmusmc.nl

^3^ Department of Health Sciences, Amsterdam Public Health Research Institute, VU University Amsterdam, De Boelelaan 1085, 1081 HV Amsterdam, The Netherlands. E-mail: carry.renders@vu.nl

^4^ Department of Medical Statistics and Bioinformatics, Leiden University Medical Center, P.O. Box 9600, 2300 RC Leiden, The Netherlands.

*Corresponding author:

Marieke Welten, MSc, Department of Epidemiology and Biostatistics (Fk28), VU Medical Center, P.O. Box 7057, 1007 MB Amsterdam, The Netherlands; Phone: +31 (0) 20 4443073; E-mail: m.welten@vumc.nl

**Text S1. Additional analyses**

In this article the results from the broken stick dataset, are considered the main analyses and the reproduction of these results in the multiple imputed (MI-)dataset additional analyses. These analyses were performed to determine if the choice to apply the broken stick method to create a complete example dataset had any effect on the performance of the different methods for developing a prediction model with a longitudinal predictor.

*The MI-dataset*

To create a second example dataset with the longitudinal predictor , we reorganized the Terneuzen data around the seven specified ages, age 0 days, 3 months, 6 months, 14 months, 2 years, 3 years, and 5.5 years, using the same approach as for the outcome of BMI-SDS at 10 years. Seven BMI-SDS variables were computed by selecting the BMI measurement taken at or between the age ranges of 0 days; 10-14.5 weeks; 5-7 months; 13-15 months; 22 months-2.5 years; 2.5-3.5 years; and 5-6 years for each subject and converting it into BMI-SDS. In case a subject had more than one BMI measurement available within an age range, the one measured closest at the specific age was selected. Also, matching age variables were created for each BMI-SDS variable of a specific age range, containing the exact age of the child at the visit during which the selected BMI-SDS measurements was taken. If no BMI-SDS measurement was available within an age range, the missing value in the age variable was set equal to the specific age (i.e. 0 days, 3 months, 6 months, 14 months, 2 years, 3 years, or 5.5 years) belonging to that age range. Next, missing values in BMI-SDS were imputed using multiple imputation according to the multivariate imputation by chained equations (MICE) procedure. For the imputation we used a wide structured dataset, predictive mean matching, N=50 imputations, and N=100 iterations. This dataset is referred to as the MI-data.

See **Additional file 1** for a visual representation of the differences between the original and the two generated example datasets (broken stick data and multiple imputed data).

*Development and performance assessment of the prediction models*

The development of the prediction models and their predictive quality assessment were replicated in the multiple imputed data. The 50 imputed datasets were analysed simultaneously. Reported performance measures of the prediction models generated in the MI-dataset were the median of the 50 adjusted (Nagelkerke) R^2^- and AUC-values.[1] See **Figure S2** and **Table S1-4** for the replicated results by these analyses.

**References**

1. Marshall A, Altman DG, Holder RL, Royston P: Combining estimates of interest in prognostic modelling studies after multiple imputation: current practice and guidelines. *BMC Med Res Methodol* 2009, 9:57.

**Figure S2.** Mean BMI-SDS at ages 0 to 6 years of overweight and non-overweight children at age 10 years (MI-data)

**Table S1.** Characteristics of the population for analysis (MI-data)

|  | **multiple imputed-data**  **N=730** |
| --- | --- |
| Male, no (%) | 343 (47.0%) |
| Visit 0d Age (years) | 0.0 (0.0 ; 0.0) |
| BMI-SDS | -0.4 (1.2) |
| Visit 3m Age (years) | 0.3 (0.2 ; 0.3) |
| BMI-SDS | -0.4 (0.9) |
| Visit 6m Age (years) | 0.5 (0.4 ; 0.6) |
| BMI-SDS | -0.4 (0.9) |
| Visit 14m Age (years) | 1.2 (1.1 ; 1.2) |
| BMI-SDS | 0.00 (0.9) |
| Visit 2y Age (years) | 2.0 (1.9 ; 2.5) |
| BMI-SDS | 0.1 (1.0) |
| Visit 3y Age (years) | 3.0 (2.5 ; 3.4) |
| BMI-SDS | -0.1 (1.0) |
| Visit 5.5y Age (years) | 5.5 (5.1 ; 6.0) |
| BMI-SDS | -0.2 (0.9) |
| Visit 10y Age (years) | 9.9 (9.1 ; 10.4) |
| BMI-SDS | -0.2 (1.0) |
| Overweight, no (%) | 90 (12.3%) |

Values are expressed as the mean of the 50 imputed datasets’ mean (SD), median (95% range) or number (%) of age at visit, BMI standard deviation score (-SDS) at visit, sex, and overweight. Missing values in the original data are: age 0d (N=13); BMI-SDS 0d (N=13); age 3m (N=173); BMI-SDS 3m (N=173); age 6m (N=70); BMI-SDS 6m (N=70); age 14m (N=532); BMI-SDS 14m (N=532); age 2y (N=401); BMI-SDS 2y (N=401); age 3y (N=156); BMI-SDS 3y (N=156); age 5.5y (N=328); BMI-SDS 5.5y (N=328);

**Table S2**. Predictive quality of prediction models developed using different methods to include longitudinal predictor BMI-SDS (MI-data)

|  |  | **outcome at 10y** | | | |
| --- | --- | --- | --- | --- | --- |
|  |  | **overweight** | | **BMI-SDS** | |
| **Method** | **Model includes** | Nk R^2^ | AUC | R^2^ | AUC |
| 1. All original measurements | BMI-SDS at age 0d, 3m, 6m, 14m, 2y, 3y, 5.5y | 0.237 | 0.803 | 0.321 | 0.800 |
| 2. Single ‘best’ measurement | BMI-SDS at age 5.5y | 0.224 | 0.790 | 0.305 | 0.790 |
| 3. Summary measurement | Mean (BMI-SDS at age 0d, 3m, 6m, 14m, 2y, 3y, 5.5y) | 0.176 | 0.769 | 0.235 | 0.769 |
| 3. Summary measurement | Maximum (BMI-SDS at age 0d, 3m, 6m, 14m, 2y, 3y, 5.5y) | 0.148 | 0.744 | 0.197 | 0.744 |
| 4. Change between measurements | BMI-SDS at age 0d and BMI-SDS changes*  between ages 3_m_-0_d_, 6_m_-3_m_ , 14_m_-6_m_ , 2_y_-14_m_ , 3_y_-2_y_ , 5.5_y_-3_y_ | 0.202 | 0.786 | 0.282 | 0.780 |
| 5. Conditional measurements | BMI-SDS at age 0d and  conditional BMI-SDS at age 3m, 6m, 14m, 2y, 3y, 5.5y | 0.237 | 0.803 | 0.321 | 0.800 |
| 6. Growth curve parameters | Mean and regression coefficients of the cubic growth curve  ($mean, b_{age}, b_{{age}^{2}}, b_{{age}^{3}}$) | 0.225 | 0.791 | 0.304 | 0.791 |

Values are the explained variance of each prediction model developed in the multiple (N=50) imputed dataset expressed as the median of the adjusted Nagelkerke R^2^ (Nk R^2^) or adjusted R^2^ (R^2^) and the Area Under the Curve (AUC). The models predicting the dichotomous outcome overweight no/yes were analysed using logistic regression. The prediction models predicting the continuous outcome BMI-SDS at age 10 were analysed using linear regression.

*Because there were slight differences in age of BMI-SDS measurements between persons in the MI-dataset the changes in this dataset were calculated as the change rate in BMI-SDS over time i.e. the change in BMI-SDS divided by the period of time this change occurred in.

|  | **outcome overweight at 10y** | | | | **outcome BMI-SDS at 10y** | | | |
| --- | --- | --- | --- | --- | --- | --- | --- | --- |
|  | BS-data | | MI-data | | BS-data | | MI-data | |
| **BMI-SDS at age** | Nk R^2^ | AUC | Nk R^2^ | AUC | R^2^ | AUC | R^2^ | AUC |
| 0d | 0.039 | 0.622 | 0.021 | 0.585 | 0.053 | 0.622 | 0.031 | 0.585 |
| 3m | 0.042 | 0.634 | 0.033 | 0.620 | 0.065 | 0.634 | 0.053 | 0.620 |
| 6m | 0.087 | 0.696 | 0.069 | 0.678 | 0.089 | 0.696 | 0.073 | 0.678 |
| 14m | 0.083 | 0.688 | 0.080 | 0.682 | 0.131 | 0.688 | 0.112 | 0.682 |
| 2y | 0.132 | 0.739 | 0.100 | 0.715 | 0.216 | 0.739 | 0.166 | 0.715 |
| 3y | 0.199 | 0.785 | 0.137 | 0.737 | 0.300 | 0.785 | 0.212 | 0.737 |
| 5.5y | 0.230 | 0.799 | 0.224 | 0.790 | 0.329 | 0.799 | 0.305 | 0.790 |

**Table S3.** Predictive quality of prediction models developed according to the method of a single ‘best’ measurement

Values are the explained variance expressed in adjusted Nagelkerke R^2^ (Nk R^2^) or adjusted R^2^ (R^2^) and the Area Under the Curve (AUC) of each prediction model developed in the broken stick dataset (BS-data) and multiple (N=50) imputation dataset (MI-data). For the MI-data the median of the R^2^, Nk R^2^ and AUC are reported. Each prediction model was build using a single variable: one of the BMI-SDS measures taken between ages 0 to 5.5years. The models predicting the dichotomous outcome overweight no/yes were analysed using logistic regression. The models predicting the continuous outcome BMI-SDS at age 10 were analysed using linear regression.

**Table S4.** Predictive quality of prediction models developed according to the growth curve method

|  | **outcome overweight at 10y** | | | | **outcome BMI-SDS at 10y** | | | |
| --- | --- | --- | --- | --- | --- | --- | --- | --- |
|  | BS-data | | MI-data | | BS-data | | MI-data | |
| **Growth curve ( BMI-SDS regressed against age) coefficients** | Nk R^2^ | AUC | Nk R^2^ | AUC | R^2^ | AUC | R^2^ | AUC |
| $mean, b_{age}$ | 0.201 | 0.786 | 0.216 | 0.790 | 0.299 | 0.785 | 0.303 | 0.790 |
| $mean, b_{age}, {se}_{age}$ | 0.210 | 0.786 | 0.218 | 0.791 | 0.314 | 0.785 | 0.305 | 0.790 |
| $mean, b_{age}, b_{{age}^{2}}$ | 0.235 | 0.800 | 0.232 | 0.796 | 0.336 | 0.801 | 0.317 | 0.795 |
| $mean, b_{age}, b_{{age}^{2}}, {se}_{{age}^{2}}$ | 0.233 | 0.802 | 0.232 | 0.797 | 0.335 | 0.801 | 0.316 | 0.795 |
| $mean, b_{age}, b_{{age}^{2}}, b_{{age}^{3}}$ | 0.241 | 0.803 | 0.225 | 0.791 | 0.337 | 0.803 | 0.304 | 0.791 |
| $mean, b_{age}, b_{{age}^{2}}, b_{{age}^{3}}, {se}_{{age}^{3}}$ | 0.239 | 0.803 | 0.223 | 0.792 | 0.336 | 0.803 | 0.303 | 0.789 |

Values are the explained variance expressed in adjusted Nagelkerke R^2^ (Nk R^2^) or adjusted R^2^ (R^2^) and the Area Under the Curve (AUC) of each prediction model developed in the broken stick dataset (BS-data) and multiple (N=50) imputation dataset (MI-data). For the MI-data the median of the R^2^, Nk R^2^ and AUC are reported. The prediction models were built with the mean of all measurements and the regression coefficients of a linear growth curve ($b_{int}, b_{age}$), a quadratic growth curve ($b_{int}, b_{age}, b_{{age}^{2}}$), and a cubic growth curve ($b_{int}, b_{age}, b_{{age}^{2}}, b_{{age}^{3}}$). Next, all three prediction models were extended with the standard error of the regression coefficients (${se}_{age},{se}_{{age}^{2}}, or {se}_{{age}^{3}}$) reflecting fluctuation around the growth curve. The models predicting the dichotomous outcome overweight no/yes were analysed using logistic regression. The models predicting the continuous outcome BMI-SDS at age 10 were analysed using linear regression.
